# Assessing the Evidence for Nonobstetric Risk Factors for Deformational Plagiocephaly: Systematic Review and Meta-Analysis

**DOI:** 10.2196/55695

**Published:** 2024-09-18

**Authors:** Christopher Robert Timothy Hillyar, Natalie Bishop, Anjan Nibber, Frances Jean Bell-Davies, Juling Ong

**Affiliations:** 1 Oxford Medical School Green Templeton College University of Oxford Oxford United Kingdom; 2 UCL Medical School University College London London United Kingdom; 3 Department of Paediatrics Wexham Park Hospital Frimley Health NHS Foundation Trust Wexham United Kingdom; 4 Craniofacial Unit Great Ormond Street Hospital for Children NHS Foundation Trust London United Kingdom

**Keywords:** deformational plagiocephaly, plagiocephaly, flat head syndrome, back to sleep, meta-analysis, systematic review, meta-analyses, systematic reviews, vitamin D, vit D, head position preference, head position, head positioning, bottle feeding, tummy time, sleeping position, motor milestones, obesity, maternal education level, male sex, formula feeding, macrocephaly, head circumference, mechanical ventilation, pediatric, pediatrics, paediatric, paediatrics, infant, infants, infancy, baby, babies, neonate, neonates, neonatal, toddler, toddlers, child, children

## Abstract

**Background:**

Plagiocephaly is defined as an asymmetrical distortion of the skull, resulting in an oblique trapezoid or parallelogram head shape. Deformational plagiocephaly (DP) is caused by forces acting on one side of the back of the head, distorting normal skull symmetry.

**Objective:**

The aims of this systematic review and meta-analysis were to critically assess the evidence for nonobstetric risk factors for DP and to make evidence-based recommendations for reducing the prevalence of DP.

**Methods:**

The selection criterion was studies reporting risk factors for DP. Case reviews, case series, expert opinions, and systematic reviews were excluded. PubMed and Web of Science were searched from August 21, 2010, to August 21, 2022. Publication bias was assessed using funnel plots. Meta-analyses were presented using forest plots.

**Results:**

A total of 19 studies (cohort studies: n=13, 68%; case-control studies: n=5, 26%; and cross-sectional studies: n=1, 5%) with a total of 14,808 participants were included. Of the 43 investigated potential nonobstetric factors, 16 (37%) were associated with DP. Of these 16 factors, 12 (75%) had odds ratios (ORs) with 95% CIs not crossing 1: insufficient vitamin D intake (OR 7.15, 95% CI 3.77-13.54), head position preference (OR 4.75, 95% CI 3.36-6.73), bottle-only feeding (OR 4.65, 95% CI 2.70-8.00), reduced tummy time (OR 3.51, 95% CI 1.71-7.21), sleeping position (OR 3.12, 95% CI 2.21-4.39), fewer motor milestones reached by the age of 6 months (OR 2.56, 95% CI 1.66-3.96), obesity (OR 2.45, 95% CI 1.02-5.90), maternal education level (OR 1.66, 95% CI 1.17-2.37), male sex (OR 1.51, 95% CI 1.07-2.12), formula feeding (OR 1.51, 95% CI 1.00-2.27), head circumference (OR 1.22, 95% CI 1.06-1.40), and mechanical ventilation (OR 1.10, 95% CI 1.00-1.14). No evidence of publication bias was detected.

**Conclusions:**

This study provides a comprehensive assessment of the nonobstetric factors associated with DP and presents 11 evidence-based recommendations for reducing its prevalence. The primary limitation is that only publication bias was assessed.

**Trial Registration:**

PROSPERO CRD42020204979; https://www.crd.york.ac.uk/prospero/display_record.php? ID=CRD42020204979

## Introduction

### Background

Plagiocephaly is defined as an asymmetrical distortion of the skull resulting in an oblique trapezoid or parallelogram head shape when viewed from the vertex position in the axial plane [1]. The severity of skull asymmetry can range from minimal focal flattening on one side of the cranial vault to severe deformation affecting the entire cranial vault, skull base, and facial skeleton. Plagiocephaly arises via 2 main mechanisms: premature fusion of ≥1 of the cranial sutures (craniosynostosis) or external mechanical forces acting on the cranial vault, which results in a distortion of the normally symmetric craniofacial skeleton (deformational plagiocephaly [DP]).

In craniosynostotic plagiocephaly involving any of the paired coronal or lambdoid sutures, the restriction of skull vault growth occurs perpendicular to the fused suture (Virchow’s law) [2,3]. Isolated craniosynostosis involving premature fusion of a single coronal suture results in an anterior plagiocephaly with brow retrusion on the affected side; similarly, craniosynostosis of a single lambdoid suture will restrict posterior cranial growth on the same side. The asymmetry is often accentuated as the remaining unfused sutures expand to enable accommodation of the rapidly growing infant brain. The majority of patients with craniosynostosis do not have an identifiable genetic cause, but this proportion is increased in patients with >1 suture involved [2,3].

Alternatively—and far more commonly—plagiocephaly is caused by deformational forces acting on one side of the back of the head, which distorts the normal symmetry of the skull in the absence of skull growth restriction due to craniosynostosis [4]. This deformity is characterized by a parallelogram-type deformity. This appears clinically as mild to severe occipital flattening, with or without ipsilateral anterior shift of the ear and orbital involvement [5]. The flattening of the posterior neurocranium, resulting from the external forces applied to the head, has led to the condition also being referred to as “flat head syndrome” [6]. The importance of external forces can be seen in the close relationship between DP and sleeping position [7], among other factors that may influence external head forces [8-12].

### Objectives

Since the 1980s, “back to sleep” campaigns have successfully publicized the benefits of supine sleeping for reducing the risk of sleep-related death, including sudden infant death syndrome [13]. Although these campaigns reduced the incidence of sudden infant death syndrome by 40%, an undesirable consequence has been an increase in the referrals of cases of DP, leading to more referrals to specialist centers [14,15]. The majority of cases of DP will resolve without intervention, and surgical treatment is not required [16]. However, in a subset of children, DP persists, even into teenage years [17]. Although physiotherapy and helmet therapy may play a role in improving head shape and limiting other long-term effects [18,19], understanding the factors that increase the risk of DP may help to prevent DP from developing. The aims of this systematic review and meta-analysis were to critically assess the evidence for risk factors for DP and to make evidence-based recommendations for reducing the prevalence of DP. This study was previously presented as a meeting abstract at the Royal College of Paediatrics and Child Health Conference on June 15, 2021.

## Methods

The study protocol, analysis, and reporting were conducted in line with the PRISMA (Preferred Reporting Items for Systematic Reviews and Meta-Analyses) 2020 guidelines [20-22]. The protocol was registered with PROSPERO (CRD42020204979).

### Search Strategy

A search of PubMed and Web of Science was performed covering the period from August 21, 2010, to August 21, 2022 (this included an initial search from August 21, 2010, to August 20, 2020, and an update search from August 21, 2010, to August 20, 2022, to ensure that the list of included studies was as up to date as possible). The combination of PubMed and Web of Science provides >97.5% coverage of published literature [23,24]. To balance comprehensive coverage with a pragmatic approach to ensure that the study was completed with limited available resources, additional databases were not searched, and hand searching and gray literature searches were also not performed. The search terms included “plagiocephaly” AND “risk factor.”

### Study Eligibility

Study titles and abstracts were screened and assessed for relevance by a single reviewer (CRTH, NB, or AN). Specifically, original studies were included if they assessed risk factors for DP. Studies in a non-English language, those involving nonhuman subjects, and low-quality or nonoriginal studies (meeting abstracts, reviews, case series, case reports, and editorials) were excluded. Duplicates were identified by assessing study titles and removed manually by CRTH. Full-text review was performed after screening by a single reviewer (CRTH, NB, or AN). Studies not reporting nonobstetric risk factors for DP were excluded. Studies reporting preventive measures such as tummy time were included. However, studies involving treatments such as physical therapy and helmet therapy were not included. As screening was performed by a single reviewer, there were no discrepancies to be resolved.

### Data Extraction and Reporting

Data extraction and reporting followed the PRISMA guidelines. However, due to resource limitations, data from eligible full-text articles were extracted by a single reviewer (CRTH, NB, or AN). The main outcomes of interest were odds ratios (ORs) and risk ratios (RRs) with 95% CIs, or significant associations, for risk factors for DP (or biomarkers for DP; eg, oblique diameter difference index). These outcomes were split into factors that were associated with DP and those that were not. Other items extracted from the eligible studies included authors, country of study, funding source, study design, study aims, total participants, percentage female, population assessed, age at baseline, and selection criteria.

### Meta-Analysis

A meta-analysis of ORs and RRs for factors associated with DP was performed. The inconsistency index (*Ι*^2^) and a Q statistic for chi-square significance for specific *df* were calculated to assess interstudy heterogeneity. Both fixed effects and random effects were reported. *P* values for 95% CIs were calculated. Excel (Microsoft Corp) and Prism (GraphPad Software) were used for statistical analysis.

### Funnel Plots

Publication bias was assessed using funnel plots of ORs and RRs for DP risk factors against study precision (1/SE). The Egger test for asymmetry was conducted using linear regression, with *P*<.05 indicating publication bias [25].

## Results

### Overview

**Figure 1:**
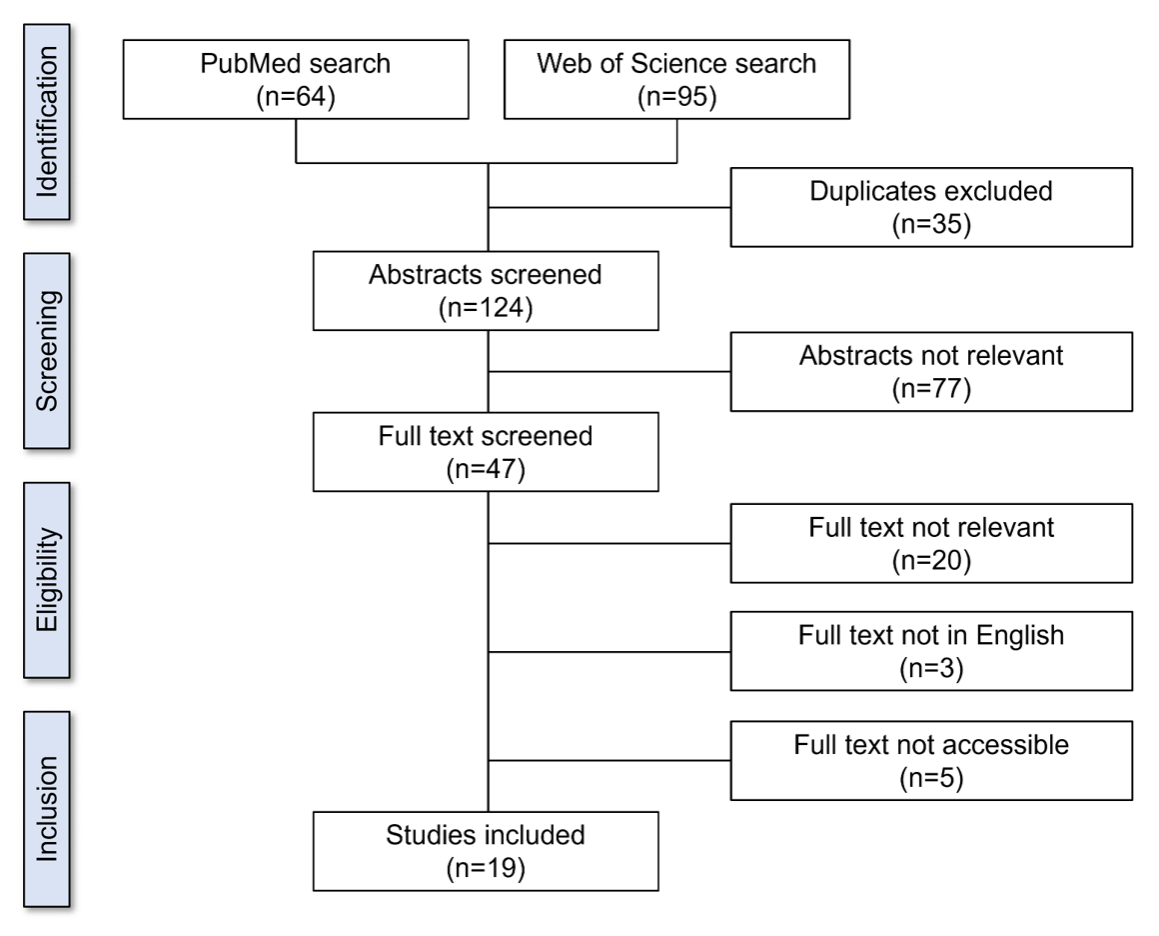
PRISMA (Preferred Reporting Items for Systematic Reviews and Meta-Analyses) 2020 flow diagram for study eligibility and inclusion.

The searches of PubMed and Web of Science yielded 159 articles; after removing 35 (22%) duplicates, 124 (78%) articles were screened based on abstract content. Of these 124 articles, 77 (62.1%) were not relevant. Full-text screening of the remaining 47 articles resulted in the exclusion of 20 (43%) irrelevant articles (these did not report nonobstetric risk factors for DP), 3 (6%) non-English articles, and 5 (11%) articles that were not accessible. Thus, of the initial 159 articles, 19 (11.9%) were eligible for inclusion in this study (Figure 1). The characteristics of the included studies are presented in Table 1.

**Table 1:** Characteristics of the included studies.

Study; country	Funding source	Study design	Study aims	Participants, n (% female)	Population assessed	Age at baseline	Selection criteria	Outcomes	Factors associated with DP^a^	Factors not associated with DP
Aarnivala et al [26]; Finland	Alma and KA Snellman Foundation and The Foundation for Pediatric Research, Finland	Retrospective cohort study	To assess risk factors for the development of DP and torticollis	155 (51)	Healthy newborns	<72 h from birth	Healthy newborns born between February 2012 and September 2013; newborns were excluded if they had chromosomal anomalies, cleft lip or palate, or craniosynostosis	ORs^b^ and RRs^c^ for risk factors for DP	NR^d^	Risk of developing DP was not associated with torticollis
Aarnivala et al [27]; Finland	Alma and KA Snellman Foundation	Prospective cohort study	To assess risk factors for cranial deformation by measuring cranial asymmetry from age 3 to 12 mo using 3D stereophotogrammetry	99 (47)	Healthy newborns	Recruited at birth	Healthy newborns included at birth residing within 20 minutes’ driving distance from Oulu University Hospital; infants with craniosynostosis, cleft lip and palate, or syndromic features were excluded	ORs and RRs for risk factors for DP	DP at age 6 mo associated with position preference and imbalance in head rotation at age 3 mo; at age 6 mo, DP associated with reaching fewer motor milestones at age 6 mo; at age 12 mo, DP associated with position preference at age 3 mo and imbalance in head rotation at age 3 mo and 6 mo; at age 12 mo, DP associated with fewer motor milestones and spending more time supine on the floor at age 6 mo; position preference at age 3 mo associated with DP at age 12 mo; at age 6 mo, DP associated with position preference at age 3 mo and fewer motor milestones reached at age 6 mo; at age 12 mo, DP associated with position preference at age 3 mo	Position preference at age 6 mo was not associated with DP at age 12 mo; there was no association between DP at age 12 mo and imbalance in head rotation at age 3 mo; no association with DP was also observed for primary sleeping position, time spent in carrier, bouncers, or car seats, time spent prone on the floor, pacifier use, illness history (acute otitis media and conditions requiring prolonged hospitalization), and duration of full breastfeeding
Ballardini et al [28]; Italy	Pediatric department of the University of Ferrara in Ferrara, Italy	Prospective cohort study	To assess prevalence and risk factors for DP in full-term infants	283 (53)	Healthy newborns	All infants: mean 11.6 (range 9.4-12.9) wk; infants with DP: mean 11.7 (range 10.0-12.8) wk; infants without DP: mean 11.6 (range 9.4-12.9) wk	Healthy infants born at term presenting at a public immunization clinic in Ferrara at age 8 to 12 wk were included; infants affected by craniosynostosis, malformations, or neurological diseases or those admitted to the NICU^e^ were excluded	ORs and RRs for risk factors for DP	Maternal and infant risk factors associated with DP included maternal age, supine sleeping position, and head position preference	Maternal and infant risk factors not associated with DP included infant sex, maternal origin, maternal education level, breast milk feeding, changing crib end, tummy time, and instruction about tummy time
Ifflaender et al [29]; Germany	The Else Kröner-Fresenius Foundation	Cross-sectional study	To assess head shape to determine the prevalence of symmetrical and asymmetrical head deformities and identify possible risk factors	195 (48)	Healthy newborns	Mean postmenstrual age: 38.4 (SD 0.9) wk	Preterm infants discharged from an intermediate care unit of a tertiary neonatal clinic in Dresden from April 2011 to January 2013 were included; all infants were included that were present on the ward at the time of measurement; those with peripheral cannula at the scalp or requiring supplemental oxygen, were excluded	ORs and RRs for risk factors for DP	Cranial vault asymmetry index ([diagonal A – diagonal B and diagonal A] × 100, where diagonal A >diagonal B) at term-equivalent age was higher (4.1%, IQR 1.9%-6.5%) in very preterm infants compared to late preterm infants and term infants; moderate or severe DP at term-equivalent age was associated with intracranial hemorrhage in preterm infants; duration of total respiratory support was higher in cases of DP compared to controls; duration of continuous positive airway pressure therapy was longer in cases of DP than in controls	No association with DP was observed for sex, bronchopulmonary dysplasia, necrotizing enterocolitis, and duration of intermittent mandatory ventilation
Kim et al [30]; South Korea	NR	Case-control study	To determine risk factors for DP and its severity, including the association between DP and infant obesity as defined by BMI	135 (40)	Infants with cranial deformation on physical examination, with age- and sex-matched controls randomly selected from the national health screening system for infants and children (confirmed to have no cranial deformation on physical examination)	2-12 mo	Infants aged 2-12 mo at the time of diagnosis of DP were included; infants with neuroimaging results positive for craniosynostosis were excluded; infants with insufficient clinical data, congenital muscular torticollis, congenital anomalies (eg, craniofacial or chromosomal anomalies), or birth injury were also excluded; age- and sex-matched controls (confirmed to have no cranial deformation on physical examination) were randomly selected from the national health screening system	ORs and RRs for risk factors for DP	Factors associated with DP included bottle-only feeding, reduced tummy time when awake, delayed motor development, and obesity at diagnosis	Factors not associated with DP included; infant male sex; maternal age; macrocephaly at birth; and macrocephaly, underweight, or overweight at the time of diagnosis
Leung et al [31]; Australia	NR	Prospective cohort study	To assess association between DP and head orientation or head strength	94 (59)	Healthy newborns	Mean 21.40 (SD 2.29) d	Healthy newborns, born at full term (37-42 wk) between June 2011 and July 2013; infants were excluded for reasons related to low birth weight (<2500 g), congenital muscular torticollis, craniosynostosis, neurological insult, or other medical or orthopedic conditions	Correlation between risk factor severity and DP severity	DP at age 9 wk was associated with asymmetrical head orientation duration at age 3 wk and 6 wk; DP at age 9 wk was significantly associated with asymmetrical head orientation strength at age 3 wk and 6 wk	No association between DP and latency to turn head
Maniglio et al [32]; Italy	NR	Prospective cohort study	To assess factors associated with DP to improve screening strategies to identify infants at risk of developing severe deformation	4337 (NR)	Infants with DP and controls without DP	6-12 wk	Infants born at the San Pietro Fatebenefratelli Hospital in Rome between January 2017 and September 2018 were included; infants with congenital deformations, born before 24 wk gestation, and infants who needed long intensive care treatment were excluded	ORs for risk factors for DP	Maternal age was associated with DP	Male sex was not associated with DP
Mawji et al [33]; Canada	The Faculty of Graduate Studies at the University of Calgary in Calgary, Alberta, provided CAD $3000 (US $2670) for data collection	Prospective cohort study	To assess potential risk factors for DP in infants aged 7-12 wk in Calgary	440 (40.7)	Healthy full-term infants	7-12 wk	Healthy full-term infants (born at ≥37 wk gestation) ranging from age 7 to 12 wk who presented for immunization at a 2-mo well-child clinic in Calgary were included	ORs and RRs for risk factors for DP	Significant difference in incidence of DP in infants placed supine to sleep compared with sleep in other positions (including prone; side; or a combination of supine, prone, or side); DP also associated with head position preference (right and left), maternal education level, and supine sleep position	No association of DP with average length of time in Canada; infant feeding position; length of tummy time received; male infant sex; and mothers who had a language barrier
Nuysink et al [34]; Netherlands	NR	Prospective cohort study	To assess predictive factors for DP in infants at corrected age >6 mo	120 (45.8)	Newborns admitted to the NICU at gestational age <30 wk or birthweight <1000 g	<1 wk	Eligible infants were born in or referred to the level III neonatal intensive care unit within 1 week of birth between January 2009 and October 2010; infants born at gestational age <30 wk or birth weight <1000 g who visited the neonatal follow-up clinic were included; infants diagnosed with a disease or dysfunction leading to symptomatic asymmetry, such as a central nervous system disorder or congenital malformation, were excluded	ORs for risk factors for DP	Association between DP and mechanical ventilation and chronic lung disease grade II in the neonatal period	NR
Launonen et al [35]; Finland	The University of Oulu Scholarship Foundation, the Orthodontic Section of the Finnish Dental Association Apollonia, the Emil Aaltonen Foundation, the Alma, and KA Snellman Foundation, the Finnish Medical Foundation, and the Foundation for Pediatric Research in Finland	Case-control study	To use 3D stereophotogrammetry to assess cranial growth, molding, and incidence of DP in preterm children compared to term-born children	68 (32)	Healthy newborns	Preterm (mean gestational age): 32.7 wk; term (mean gestational age): 40.0 wk	Infants were considered eligible if they had no cheilopalatoschisis, craniosynostosis, or dysmorphic features and if they resided within Oulu region, Finland; all participants were born between 2012 and 2015 at Oulu University Hospital; the control group was randomly selected from a previously collected nonintervention cohort by computer-based random selection and matched for sex	OCLR^f^ mean difference	NR	No difference in head shape (OCLR) between preterm and full-term children or between sexes
Pogliani et al [36]; Italy	NR	Retrospective cohort study	To assess risk factors for DP at birth	413 (50)	Healthy newborns	<72 h from birth	Neonates at gestational age >33 wk born between May 2011 and January 2012; newborns with extreme low birth weight or presenting with major congenital malformations needing NICU transfer were excluded; children of mothers with a documented TORCH^g^ infection were also excluded	ORs and RRs for risk factors for DP	Association between DP and male sex	NR
Roberts et al [37]; United Kingdom	NR	Retrospective cohort study	To test the hypothesis that ventriculoperitoneal shunt insertion significantly increases contralateral DP	339 (42)	Children aged 0-16 y with ventriculoperitoneal shunts	NR	Children aged 0-16 y with at least 1 follow-up scan from the surgical database at the pediatric neurosurgery department of Birmingham Children’s Hospital between 2006 and 2013; children without imaging were excluded	ORs and RRs for risk factors for DP	DP was associated with occipital shunt placement; a statistically significant difference in the probability of becoming plagiocephalic between neonates and children aged 12-16 y was observed; boys were more likely to develop shunt-associated plagiocephaly than girls	No difference in DP between neonates and infants, neonates and children aged 3-5 y, neonates and children aged 1-3 y, and neonates and children aged 5-12 y
Sheu et al [38]; United States	Cooperative agreement from the Centers for Disease Control and Prevention (U01DD000494) and Title V Maternal and Child Health Block Grant funds from the Health Resources and Services Administration	Retrospective cohort study	To assess factors that may explain a 9-fold increase in plagiocephaly in Texas from 1999 to 2007	6295 (38)	Infants with DP	NR	Cases identified using the Texas Birth Defects Registry with a definite diagnosis of DP (British Paediatric Association code 754.050), born between January 1, 1999, and December 31, 2007; cases of plagiocephaly with craniosynostosis were excluded	Mean difference or percentage difference for risk factors for DP	Lower maternal education level was associated with DP	No association of DP with maternal age or race and ethnicity, infant sex; mean age at DP diagnosis did not significantly change over time
Solani et al [39]; Iran	Grant funding from the Vice-Chancellor for Research, Kashan University of Medical Sciences, Kashan, Iran	Case-control study	To determine the risk factors of positional plagiocephaly in healthy infants	300 (NR)	Healthy Iranian infants	8-12 wk	Healthy full-term (gestational age >37 wk) infants aged 8-12 wk who were referred to the pediatric neurology clinic at Shahid Beheshti Hospital in Kashan, affiliated with Kashan University of Medical Sciences, were included	ORs for risk factors for DP	Factors associated with DP included male sex, head circumference, and supine sleeping position	Firmness of headrest was not associated with DP
Tang et al [40]; United States	NR	Prospective cohort study	To assess the prevalence of DP in infants with NBPP^h^ and spontaneous recovery from DP	28 (50)	Full-term infants aged >3 mo and <1 y with NBPP	Mean 3 (SD 3) mo	Full-term infants aged >3 mo and <1 y with NBPP; infants with neurological or congenital comorbidities in addition to NBPP, helmet therapy for plagiocephaly, and surgical procedures related to NBPP were excluded; infants with craniosynostosis were also excluded	Mean difference or percentage difference for risk factors for DP	NR	Maternal age and race (Black or White) were not associated with DP
Valkama et al [41]; Finland	NR	Case-control study	To assess the prevalence of DP in children with DDH^i^	120 (56)	Children with DDH with or without DP	Children with DDH: mean 8.0 (SD 1.4) y; matched controls: mean 7.9 (SD 1.3) y	Children with DDH from among newborn infants born at the Oulu University Hospital in Oulu, Finland, were included; preterm children and children with disabilities were excluded	ORs and RRs for risk factors for DP	10.3% of the children with DDH and only 1.5% of the control children had DP	OCLR was equal between children with DDH and controls; no association between side of DDH and DP
Van Vlimmeren et al [42]; Netherlands	Grant from the Scientific Committee of The Royal Dutch Association for Physiotherapy (BU002/10)	Prospective cohort study	To assess skull shape in healthy newborns until age 5.5 y in children with position preference at 7 wk and those without and in children with position preference who received pediatric physical therapy intervention and those who did not	380 (53)	Healthy newborns	<48 h from birth	Healthy newborns (gestational age ≥36 wk) born between December 2004 and September 2005 at Hospital Bernhoven; children with congenital muscular torticollis (Kaplan type 2 and 3), dysmorphism, or syndromes were excluded	Mean difference or percentage difference for risk factors for DP	Association between DP and position preference	No association between potential risk factors (nursing, feeding, sleeping, and playing positioning habits) at age 7 wk and skull deformity at age 24 mo and 5.5 y; a trend toward significance between time spent playing prone (tummy time) at age 7 wk and the ODDI^j^ percentage at age 24 mo
Weernink et al [43]; Netherlands	ZonMw, the Netherlands Organization for Health Research and Development (170.992.501)	Case-control study	To assess the influence of adherence to recommendations for vitamin D supplement intake of 10 μg/d (400 IU) in the first months of life (child) on the occurrence of DP of the child at age 2-4 mo	823 (46)	Infants with DP and those without	2-4 mo	Children born between November 22, 2009, and June 9, 2010, with mild to severe DP from the Helmet Therapy Assessment in Deformed Skulls study; controls were included from a 2010 survey on infant milk feeding	ORs and RRs for risk factors for DP	Insufficient vitamin D supplement intake during early infancy was associated with DP; maternal sociodemographic factors associated with DP included mother’s age and mother’s education level; infant factors associated with DP included male sex, formula feeding, and milk formula consumption after birth	Maternal sociodemographic factors not significantly associated with DP included mother’s country of birth (other than the Netherlands); infant factors not associated with DP included time child spent outdoors
Van Cruchten et al [9]; Netherlands	NR	Cohort study	To assess the impact of risk factors on the type and severity of DP	184 (30.4)	Infants seen at outpatient clinic with a parental concern for DP	3-14 mo	Exclusion criteria consisted of children aged >14 mo or <3 mo and other forms of cranial deformation, such as cranial synostosis	Differences in ODDI for risk factors for DP	Negative correlation between age and ODDI; positive correlation between ODDI and position preference right and position preference left	No association between ODDI and developmental delay, family history of DP, sex, and torticollis

^a^DP: deformational plagiocephaly.

^b^OR: odds ratio.

^c^RR: risk ratio.

^d^NR: not reported.

^e^NICU: neonatal intensive care unit.

^f^OCLR: oblique cranial length ratio.

^g^TORCH: toxoplasmosis, other (including infections such as syphilis, varicella-zoster, and parvovirus B19), rubella, cytomegalovirus, and herpes simplex virus.

^h^NBPP: neonatal brachial plexus palsy.

^i^DDH: developmental dysplasia of the hip.

^j^ODDI: oblique diameter difference index.

### Demographic Factors

#### Age

The study by van Cruchten et al [9] reported a negative correlation between age and oblique diameter difference index (a biomarker for DP). However, the study by Sheu et al [38] (which assessed factors associated with a 9-fold increase in plagiocephaly between 1999 and 2007 in Texas, United States) reported no association between age and DP. Finally, the study by Roberts et al [37], which assessed DP in children with ventriculoperitoneal shunts, reported that being aged 12 to 16 years at the time of shunt insertion was associated with DP.

#### Sex

**Figure 2:**
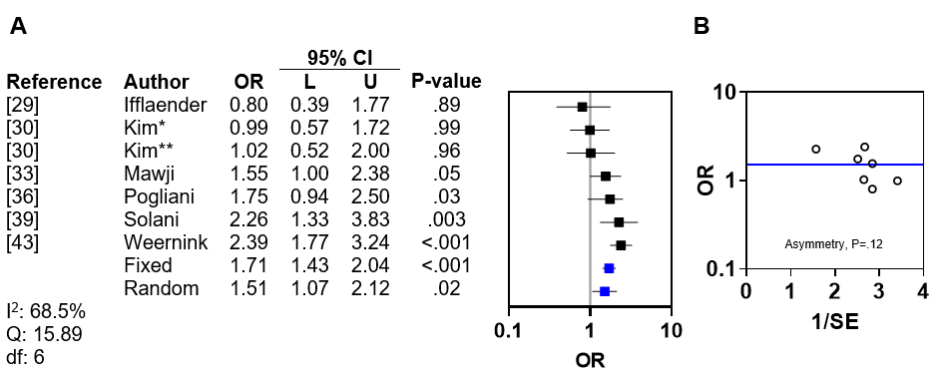
Meta-analysis and funnel plot for male sex. (A) Forest plot of odds ratios for deformational plagiocephaly related to male sex with fixed and random effects. (B) Funnel plot with linear regression test of asymmetry. The blue line indicates random effects. *Mild to moderate deformational plagiocephaly (DP; univariate analysis); **severe DP (univariate analysis).

Of the 19 studies, 4 (21%) demonstrated an association between male sex and DP [33,36,39,44], 1 (5%) reported borderline association [37], and 7 (37%) reported no association between male sex and DP [9,28-30,32,35,38]. Of these 12 studies, 6 (50%) [29,30,33,36,39,43] reported ORs, and a meta-analysis of these ORs revealed interstudy heterogeneity (*I*^2^=68.5%; Q statistic=15.89; *df*=6). The pooled fixed and random effects ORs for DP related to male sex were 1.71 (95% CI 1.43-2.04; *P*<.001) and 1.51 (95% CI 1.07-2.12; *P*=.02), respectively (Figure 2A [29,30,33,36,39,43]). Asymmetry analysis of the funnel plot excluded publication bias (*P*=.12; Figure 2B).

#### Race

Race was investigated by Tang et al [40], who included a cohort of children with brachial plexus palsy. In this group, race was not associated with DP that developed after brachial plexus injury.

### Developmental Factors

#### Developmental Delay

Developmental delay was investigated as a risk factor for DP by van Cruchten et al [9], who concluded that developmental delay was not associated with DP.

#### Developmental Dysplasia of the Hip

Developmental dysplasia of the hip (DDH) was investigated as a risk factor for DP by Valkama et al [41], who assessed the prevalence of DP in children with DDH. DDH was associated with DP compared to controls without DDH.

#### Head Circumference

Head circumference was investigated by Solani et al [39], who concluded that it was associated with DP (OR 1.22, 95% CI 1.06-1.40).

#### Motor Milestones

**Figure 3:**
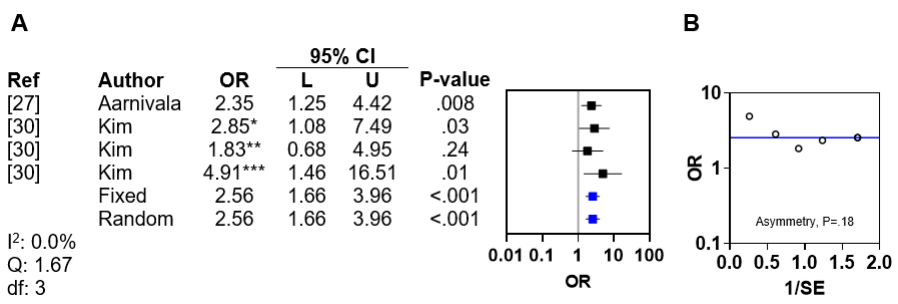
Meta-analysis and funnel plot for reaching fewer motor milestones by age 6 months. (A) Forest plot of odds ratios for deformational plagiocephaly related to reaching fewer motor milestones by age 6 months with fixed and random effects. (B) Funnel plot with linear regression test of asymmetry. The blue line indicates fixed effects. *Adjusted OR (aOR) for deformational plagiocephaly (DP; multivariate analysis); **odds ratio (OR) for mild to moderate DP (only univariate analysis available); ***aOR for severe DP (multivariate analysis).

Reaching fewer motor milestones by age 6 months was investigated as a risk factor by 2 (11%) of the 19 included studies [27,30]. The first study reported that reaching fewer motor milestones by age 6 months was associated with DP (adjusted OR [aOR] 2.35, 95% CI 1.25-4.42) [27]. The second study found an association between delay in motor development and DP [30]. A meta-analysis of the ORs from these studies identified no interstudy heterogeneity (*I*^2^=0%; Q statistic=1.67; *df*=3; Figure 3A [27,30]). The pooled fixed and random effects ORs for DP related to delayed motor milestones were both 2.56 (95% CI 1.66-3.96; *P*<.001). Asymmetry analysis of the funnel plot excluded publication bias (*P*=.18; Figure 3B).

#### Overweight and Underweight

Being overweight at diagnosis of DP was investigated as a risk factor for DP by Kim et al [30], who reported that being overweight at diagnosis was not associated with DP. The same study reported that being underweight at diagnosis was also not associated with DP.

### Dietary Factors

#### Bottle-Only Feeding

Bottle-only feeding was investigated by Kim et al [30], who demonstrated that bottle-only feeding was associated with DP (aOR 4.65, 95% CI 2.70-8.00).

#### Breast Feeding

The duration of exclusive breast feeding was investigated by Aarnivala et al [27], who demonstrated that the duration of exclusive breast feeding was not associated with DP.

#### Formula Feeding

Formula feeding was investigated by Weernink et al [43], who reported that children who developed DP by age 2 to 4 months were more likely to be formula fed (aOR 1.51, 95% CI 1.00-2.27).

#### Vitamin D Intake

Vitamin D intake in infants was investigated by Weernink et al [43], who reported that children who developed DP by age 2 to 4 months were more likely to have insufficient vitamin D intake (aOR 7.15, 95% CI 3.77-13.54).

### Maternal Factors

#### Maternal Age

Of the 19 included studies, 3 (16%) demonstrated an association between maternal age and DP [28,32,43]. However, 3 (16%) of the 19 studies reported no association [30,38,40]. Only 1 (17%) of these 6 studies reported an OR indicating that increased maternal age was a protective factor for the development of DP (OR 0.94, 95% CI 0.91-0.97) [43].

#### Maternal Education Level

**Figure 4:**
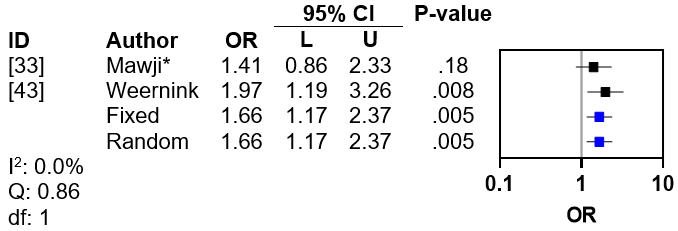
Meta-analysis for maternal education level. *Reciprocal odds ratio (OR) for postsecondary education.

Of the 19 included studies, 3 (16%) demonstrated an association between maternal education level and DP [33,38,43], and 1 (5%) reported no significant association [28]. Only 2 (67%) of the 3 studies reported ORs [33,43]. Of these 2 studies, 1 (50%) indicated that low maternal education level was an adverse factor in the development of DP (aOR 1.97, 95% CI 1.19-3.26) [43], while 1 (50%) suggested that postsecondary education was not a protective factor (OR 0.71, 95% CI 0.43-1.16) [33]. The OR from the former was used in a meta-analysis with the inverse OR from the latter, which demonstrated no interstudy heterogeneity (*I*^2^=0%; Q statistic=0.86; *df*=1; Figure 4 [33,43]). The pooled fixed effects OR for DP related to low education level was 1.66 (95% CI 1.17-2.37; *P*<.005).

#### Maternal Language Barriers

The study by Ballardini et al [28] investigated whether infants with mothers who experience a language barrier when receiving medical advice had a higher rate of DP. The study suggested that maternal language barrier was not associated with DP in the infant.

#### Maternal Race and Country of Origin

Of the 19 included studies, 3 (16%) investigated whether DP was associated with maternal race and country of origin [28,38,43]. All 3 studies suggested that maternal race and country of origin was not associated with DP in the infant.

#### Length of Time in the Country of Study

The study by Mawji et al [33] investigated whether the length of time spent by mothers in the country in which the study was conducted was associated with DP. The study reported that length of time in the country of study was not associated with DP.

#### Pacifier Use

Pacifier use by mothers in healthy infants was investigated as a risk factor for DP by Aarnivala et al [27], who reported that pacifier use was not associated with DP.

#### Tummy Time Instructions

The study by Ballardini et al [28] investigated whether DP was associated with mothers receiving instructions about tummy time. The study reported that receiving instructions about tummy time was not associated with DP.

### Medical and Surgical Factors

#### Bronchopulmonary Dysplasia

Bronchopulmonary dysplasia was investigated as a risk factor for DP by Ifflaender et al [29], who included a cohort of infants born prematurely. The study reported that bronchopulmonary dysplasia was not associated with DP.

#### Chronic Lung Disease

Chronic lung disease grade II was investigated by Launonen et al [34], who also assessed DP risk factors in infants born prematurely. The study reported that chronic lung disease grade II was associated with DP.

#### Family History of DP

Family history of DP was investigated by van Cruchten et al [9]. The study suggested that family history of DP was not associated with DP.

#### History of Illness

History of illness was investigated by Aarnivala et al [27]. The study suggested that history of illness was not associated with DP.

#### Intracranial Hemorrhage

Intracranial hemorrhage was investigated by Ifflaender et al [29], who assessed DP risk factors in infants born prematurely. The study reported that intracranial hemorrhage was not associated with DP.

#### Macrocephaly

Macrocephaly at birth was investigated by Kim et al [30]. The study suggested that macrocephaly at birth was not associated with DP. Macrocephaly at diagnosis of DP was also investigated, and it was found that this factor too was not associated with DP (OR 1.38, 95% CI 0.63-3.04) [30]. A lack of association was reported for subgroups with mild to moderate DP (OR 1.48, 95% CI 0.62-3.53) and severe DP (OR 1.19, 95% CI 0.04-3.58) [30].

#### Mechanical Ventilation

Of the 19 included studies, 2 (11%) demonstrated an association between mechanical ventilation and the development of DP in preterm infants [29,34]. Of these 2 studies, 1 (50%) reported an OR of 1.10 (95% CI 1.00-1.14) for mechanical ventilation [34]. The other study also suggested that the duration of total respiratory support (continuous positive airway pressure and intermittent mandatory ventilation) and the duration of continuous positive airway pressure alone were associated with DP, while intermittent mandatory ventilation alone was not associated with DP [29].

#### Necrotizing Enterocolitis

Necrotizing enterocolitis was investigated by Ifflaender et al [29], who included a cohort of infants born prematurely. The study reported that necrotizing enterocolitis was not associated with DP.

#### Obesity

Obesity at diagnosis of DP was investigated by Kim et al [30]. Obesity at diagnosis of DP was defined as BMI >97th percentile. The study concluded that obesity at diagnosis of DP was associated with DP (aOR 2.45, 95% CI 1.02-5.90). The study also suggested that obesity at diagnosis of DP was associated with severe DP (aOR 4.10, 95% CI 1.42-11.90) but was not associated with mild to moderate DP (aOR 2.29, 95% CI 0.86-6.05).

#### Occipital Shunt Placement

Occipital shunt placement was investigated by Roberts et al [37], who concluded that occipital shunt placement was associated with DP compared to frontal shunt placement.

#### Torticollis

Torticollis was investigated by 3 (16%) of the 19 included studies, all of which reported no association between torticollis and DP [9,26,38].

### Positional and Environmental Factors

#### Carriers, Bouncers, Car Seats, and Headrests

Time spent in carriers, bouncers, and car seats was investigated by Aarnivala et al [27], who reported that time spent in carriers, bouncers, and car seats was not associated with DP. Firmness of headrest was investigated by Solani et al [39], who reported that firmness of headrest was not associated with DP (OR 1.31, 95% CI 0.72-2.37).

#### Change of Crib End

Change of crib end (ie, alternating the infant’s sleeping position by placing their head at different ends of the crib) was investigated as a risk factor for DP by 2 (11%) of the 19 included studies [33,42]. Both studies suggested that change of crib end was not associated with DP.

#### Feeding Position

Feeding position was investigated as a risk factor for DP by 2 (11%) of the 19 included studies [33,42]; both demonstrated that feeding position was not associated with DP.

#### Latency in Head Turning

Latency in head turning was investigated by Leung et al [31], who concluded that latency in head turning was not associated with DP.

#### Playing Position

Playing position was investigated by van Vlimmeren et al [42], who demonstrated that playing position was not associated with DP.

#### Head Position Preference

**Figure 5:**
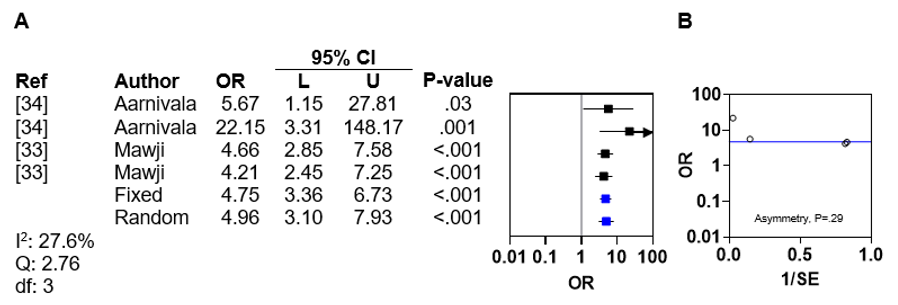
Meta-analysis and funnel plot for head position preference. (A) Forest plot of odds ratios for deformational plagiocephaly related to head position preference with fixed and random effects. (B) Funnel plot with linear regression test of asymmetry. The blue line indicates fixed effects.

Head position preference was investigated by 6 (32%) of the 19 included studies, all of which demonstrated an association between head position preference and DP [9,27,28,31,33,42]. Of these 6 studies, 2 (33%) [33,34] reported ORs, and a meta-analysis of these ORs revealed negligible interstudy heterogeneity (*I*^2^=27.6%; Q statistic=2.76; *df*=3) (Figure 5A [33,34]). The pooled fixed and random effects ORs for DP related to head position preference were 4.75 (95% CI 3.36-6.73; *P*<.001) and 4.96 (95% CI 3.10-7.93; *P*<.001), respectively. Asymmetry analysis of the funnel plot excluded publication bias (*P*=.28; Figure 5B).

#### Sleeping Position

**Figure 6:**
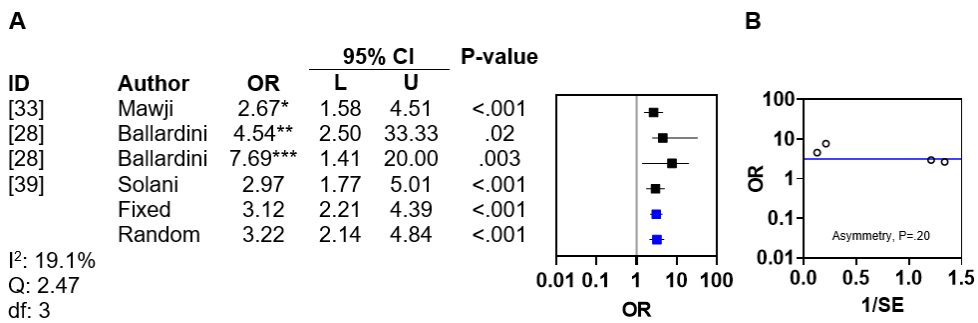
Meta-analysis and funnel plot for supine sleeping position. (A) Forest plot of odds ratios for deformational plagiocephaly related to supine sleeping position with fixed and random effects. (B) Funnel plot with linear regression test of asymmetry. The blue line indicates fixed effects. *Supine versus prone; **reciprocal prone versus supine; ***reciprocal side versus supine.

Sleeping position (supine vs prone) was investigated by 3 (16%) of the 19 included studies [28,33,39]. Of these 3 studies, 2 (67%) demonstrated an association between supine sleeping and DP [33,39], while 1 (33%) reported a protective effect from prone sleeping (OR 0.13, 95% CI 0.03-0.40) or side sleeping (OR 0.22, 95% CI 0.05-0.71) [28]. The ORs from 2 (67%) [33,39] of the 3 studies were used with the inverse OR from the third study [28] in a meta-analysis, which demonstrated negligible interstudy heterogeneity (*I*^2^=19.1%; Q statistic=2.47; *df*=3; Figure 6A [28,33,39]). The pooled fixed and random effects ORs for DP related to sleeping position were 3.12 (95% CI 2.21-4.39; *P*<.001) and 3.22 (95% CI 2.14-4.84; *P*<.001), respectively. Asymmetry analysis of the funnel plot excluded publication bias (*P*=.20; Figure 6B).

#### Time Spent Prone on the Floor

The study by Aarnivala et al [27] investigated time spent prone on the floor and reported no association with DP.

#### Tummy Time

Tummy time was investigated by 4 (21%) of the 19 included studies [28,30,33,42]. Of these 4 studies, 1 (25%) reported an association between reduced tummy time when awake and DP (aOR 3.51, 95% CI 1.71-7.21) [30]. The remaining studies (3/4, 75%) reported that tummy time was not associated with DP but provided no ORs [28,33,42].

#### Time Spent Outdoors

Time spent outdoors was investigated by Weernink et al [43], who reported no association with DP.

#### Time Spent Supine on the Floor

Time spent supine on the floor was investigated as a risk factor for DP by Aarnivala et al [27], who reported that children with DP at age 12 months spent more time supine on the floor at age 6 months.

### Summary of Nonobstetric Factors Associated With DP

A summary of 16 nonobstetric factors associated with DP is presented in Table 2. ORs are provided for 12 (75%) of these 16 nonobstetric factors.

**Table 2:** Odds ratios (ORs) for factors associated with deformational plagiocephaly. ORs are reported from single studies unless marked with a superscript (“a” or “b”) indicating pooled ORs from fixed or random effects meta-analyses.

Factors	OR (95% CI)	References
Insufficient vitamin D intake	7.15 (3.77-13.54)	[42]
Head position preference	4.75^a^ (3.36-6.73)	[33,34]
Bottle-only feeding	4.65 (2.70-8.00)	[30]
Reduced tummy time	3.51 (1.71-7.21)	[30]
Sleeping position	3.12 (2.21-4.39)	[28,33,39]
Fewer motor milestones by age 6 mo	2.56^a^ (1.66-3.96)	[27,30]
Obesity (BMI >97th percentile)	2.45 (1.02-5.90)	[30]
Maternal education level	1.66^a^ (1.17-2.37)	[33,43]
Male sex	1.51^b^ (1.07-2.12)	[29,30,33,36,43]
Formula feeding	1.51 (1.00-2.27)	[43]
Head circumference	1.22 (1.06-1.40)	[39]
Mechanical ventilation	1.10 (1.00-1.14)	[34]
Chronic lung disease grade II	NR^c^	[34]
DDH^d^	NR	[41]
Maternal age	NR	[28,32,43]
Time spent supine	NR	[27]

^a^OR from a fixed effects meta-analysis where interstudy heterogeneity was <50%.

^b^OR from a random effects meta-analysis where interstudy heterogeneity was ≥50%.

^c^NR: not reported.

^d^DDH: developmental dysplasia of the hip.

## Discussion

### Principal Findings

This study assessed evidence of association with DP for 43 nonobstetric factors (demographic factors: n=3, 7%; developmental factors: n=6, 14%; dietary factors: n=4, 9%; maternal factors: n=8, 19%; medical and surgical factors: n=11, 26%; and positional and environmental factors: n=11, 26%). Of these 43 factors, 16 (37%) were associated with DP (demographic factors [male sex]: n=1, 6%; developmental factors [DDH, head circumference, and delay in motor milestones]: n=3, 19%; dietary factors [bottle-only feeding, formula feeding, and vitamin D intake]: n=3, 19%; maternal factors [maternal age and maternal education level]: n=2, 12%; medical and surgical factors [chronic lung disease grade II, mechanical ventilation, and obesity at diagnosis of DP]: n=3, 19%; and positional and environmental factors [head position preference, sleeping position, reduced tummy time, and time spent supine on the floor]: n=4, 25%). With the notable exceptions of maternal age, mechanical ventilation, and tummy time, these associations were either supported by nonconflicting evidence or a meta-analysis that resolved conflicting evidence into an association. Of the 16 factors, 12 (75%) had ORs that ranged from 1.10 (mechanical ventilation) to 7.15 (insufficient vitamin D intake). Of the 5 factors assessed by meta-analysis (male sex, reaching fewer motor milestones by age 6 months, maternal education level, head position preference, and sleeping position), only 1 (20%; male sex) was associated with interstudy heterogeneity (≥50%). No evidence of publication bias was detected.

### Evidence-Based Recommendations

Strategies to reduce the prevalence of DP have included guidance about the infant’s environment, positioning, and handling, with the goal of creating a nonrestrictive environment that promotes spontaneous and unhindered physical movement and symmetrical motor development [27,45].

On the basis of the evidence presented in this study, the following 11 recommendations, presented in order of importance, are offered with the aim of reducing the prevalence of DP.

First, to ensure bone health in infants, it is critical that vitamin D intake is adequate [46]. Vitamin D level should be assessed regularly during development, and dietary supplementation should be considered if vitamin D level is low.

Second, during the first months of life, babies develop a head position preference [47], and this preference is more often to the right [44,48]. The increased compressive forces on one side of the head for prolonged periods causes flattening on the side being compressed. It has been proposed that the position of the fetus in the later stages of pregnancy may, in part, be responsible for position preference [28,49]. However, there is also evidence that position preference can be modified by varying head position during sleep to encourage equal distribution of pressure [50]. Other strategies to mitigate head position preference should be used, such as gently moving the infant’s head to the unfavored side when asleep or physical therapy with or without kinesiological tape to reduce tightness in the neck muscles to facilitate easier neck turning [51,52].

Third, sufficient tummy time should be provided to strengthen the infant’s head, neck, and arms and reduce time spent supine when awake.

Fourth, as infants tend to turn away from windows and toward the center of a room, it is recommended to alternate the infant’s sleeping position by placing their head at different ends of the crib. This concept is supported by 2 studies suggesting no association between crib end change and DP [33,42]. However, it is important to state that the lack of association between crib end change and DP does not necessarily mean that changing the crib end will provide an effective treatment for established DP; it may merely help to reduce the risk of DP developing.

Fifth, although bottle-only feeding should not be discouraged, it is recommended to alternate feeding positions, using both the dominant and nondominant sides when holding the infant. Bottle feeding, as opposed to exclusively breast feeding, may be positively associated with obesity (which has also been identified as a risk factor for DP) [53]. This coassociation may, in part, explain the association of bottle-only feeding with DP.

Sixth, although formula feeding should also not be discouraged, it is recommended to alternate feeding positions. It has been suggested by Weernink et al [43] that this particular risk factor is associated with lower maternal education level. Similar to bottle feeding, formula feeding, as opposed to exclusively breast feeding, may be positively associated with obesity (itself a risk factor for DP) [53].

Seventh, if motor milestones are delayed, infants should be referred for specialist assessment by a pediatrician. Similarly, obesity (BMI >97th percentile) should be identified and managed by a specialist pediatrician.

Eighth, to mitigate the impact of low maternal education level, educational resources and face-to-face education outlining factors associated with DP, as well as recommendations to reduce their impact, should be provided to all families at the 6-week postnatal infant check-up, with particular emphasis on those with low educational levels. A lower maternal education level results in worse health outcomes in infancy and later life [54]; although improving the education level of future mothers should be a national priority, there are other factors associated with low education level that may be immediately modifiable. These include access to information and access to resources [55].

Ninth, Information should be provided to families that male infants are at higher risk of DP. Losee et al [49] suggested that male infants tend to have larger, less flexible heads at birth, which are more likely to become deformed by compressive forces in utero and in the birth canal. Families should be particularly encouraged to engage with strategies to mitigate DP in male infants.

Tenth, information should be provided to families that infants with greater head circumference are at higher risk of DP. Families should be particularly encouraged to engage with strategies to mitigate DP in these infants. 

Eleventh, information should also be provided to families that infants who have had mechanical ventilation are at higher risk of DP. Families and care teams of infants requiring mechanical ventilation should be encouraged to engage with strategies to mitigate DP in these infants.

Alternative strategies for mitigating the impact of DP, such as helmet therapy, although costly for individuals and health care systems, may help avoid more invasive strategies [56]. It has been demonstrated that education provided by health care professionals, such as health visitors, midwives, and nurses, can successfully reduce the prevalence of DP [57]. Adopting these recommendations may lead to a reduction in the prevalence of DP.

### Strengths of This Study

The strengths of this study include a robust methodology. The protocol was registered with PROSPERO, and the reporting was in line with the PRISMA 2020 guidelines [22]. The databases searched provided >97.5% coverage [23]. Standardized data extraction was performed to minimize errors. Meta-analysis was performed where data allowed. Funnel plots suggested that publication bias did not impact the results; thus, a trim-and-fill analysis was not necessary to correct for asymmetry [58].

This study represents the most comprehensive analysis of nonobstetric factors associated with DP published to date. A systematic review by Bialocerkowski et al [59] only identified 5 factors associated with DP, of which 3 (60%) were nonobstetric factors (male sex, supine position, and neck problems) [59]. Another systematic review by De Bock et al [60] identified male sex, supine sleeping position, limited neck rotation, head position preference, lower level of activity, and reduced tummy time as the most important risk factors [60]. By contrast, our study provides evidence of association for 16 factors and confirms the association of male sex, supine sleeping position, head position preference, and reduced tummy time with DP.

A more recent systematic review by Inchingolo et al [61] provided only 2 recommendations to mitigate the impact of nonobstetric risk factors for DP: at least 30 minutes of tummy time and the use of a passive sleep curve mattress to improve harmonious skull growth. In addition, it did not include meta-analyses to support these recommendations. By contrast, our study makes 11 recommendations for nonobstetric factors to reduce the prevalence of DP. The systematic review by Inchingolo et al [61] also did not assess publication bias. Our review suggested that none of the recommendations were influenced by publication bias.

### Limitations of This Study

To ensure the completion of the study, additional databases were not searched, and hand searching and gray literature searches were also not performed. This inevitably limited the comprehensiveness of this study, although no systematic review can claim to be truly comprehensive because this would necessitate continual inclusion of newly published studies. The search terms were designed to be specific yet pragmatic to ensure the completion of the work with limited resources; thus, some studies may have been overlooked. Due to resource limitations, it was not possible to record more detailed reasons for the exclusion of screened articles other than not reporting risk factors for DP. Resource constraints also prevented the screening process from being conducted by 2 independent reviewers. Instead, abstract and full-text screening was performed by a single reviewer. This impacts the reproducibility of the study due to the increased potential for errors in study selection when screening is undertaken by a single reviewer. That said, because the selection criteria were clearly set out, errors due to the application of these criteria during the screening process are likely to have had minimal impact on the study outcomes. In addition, it was not feasible, due to resource limitations, to contact study authors to attempt to collect raw data if a study did not report ORs for a risk factor. This may have limited the data that could have been used in meta-analysis. If contacting authors had been feasible, more factors could potentially have been analyzed quantitively to resolve discrepancies between the included studies. This represents a significant limitation of our study.

These factors may, in part, account for the apparent disparities reported in our results compared to other literature. These disparities include the apparent lack of association between developmental delay and DP [9], while demonstrating a significant association between reaching fewer motor milestones and DP [27,30]. A recent systematic review by Martinuik et al [62] included 19 studies that assessed the association between developmental delay and DP. Notwithstanding the fact that the authors included multiple studies that used the same study population more than once, a positive association between developmental delay and DP was reported in a majority of the studies [62]. The fact that our study did not demonstrate a similar association may, in part, be due to the inclusion of studies that assessed different populations, as well as methodological limitations in our search strategy that limited the identification of studies that may have met our eligibility criteria. Another study of a large primary care cohort of 77,108 patients has provided further evidence in support of an association between developmental delay and DP [63]. That said, the literature remains conflicting, with other studies unable to demonstrate an association between presence or degree of developmental delay and DP [64]. Commentators have highlighted the fact that most studies are retrospective and observational by design, and this limits conclusions about the correlative versus causative relationship between developmental delay and DP [65].

Another disparity reported in our study is that torticollis was not associated with DP [9,26,38]. A number of literature reviews have previously reported an association between congenital muscular torticollis and DP [8,59]. Although these studies were published a decade or so ago, it logically follows that if other positional factors, such as head position preference and sleeping position, have been found in more contemporaneous studies to be significantly associated with DP [28,33,34,39], then torticollis would also be expected to be found to be associated with DP. These conflicts reported in the literature may be due to the limitations of individual study design or differences between different study populations. Therefore, we recommend interpreting with caution the lack of association between torticollis and DP reported in our study, given the lack of a mechanistic explanation that reconciles this result with other positional factors that were found to be associated with DP.

In this review, we have included a study involving patients who developed plagiocephaly after ventriculoperitoneal shunt insertion [37]. Although the authors characterized the plagiocephaly as “positional,” implying that external forces had caused the skull deformity, an alternative hypothesis is that shunt-associated plagiocephaly is a different disease entity from DP [37]. This study has been included for comprehensiveness [37], but the results should be interpreted with caution alongside those of other studies.

Finally, another limitation includes the lack of a Grading of Recommendations Assessment, Development, and Evaluation (GRADE) assessment of evidence quality, which impacts the certainty of the conclusions and recommendations set out in this study.

### Conclusions

In summary, this study provides the most comprehensive meta-analytic assessment of nonobstetric factors associated with DP published to date. It offers 11 evidence-based recommendations that can be adopted by health care systems globally to reduce the prevalence of DP. Future research should focus on investigating factors for which the literature is conflicting but quantitative data are lacking to enable meta-analyses to be performed; for example, maternal age was the only factor reported to be protective against DP, but conflicting studies reported that there was no association without providing quantitative data. Thus, maternal age as a protective factor for DP should be investigated further to provide quantitative data for meta-analytic approaches to determine its protective effect. Further research should also address the nature of specific relationships between risk factors and DP; for example, both bottle-only feeding and formula feeding have been associated with DP. Studies should focus on understanding the nature of this relationship, that is, whether this relationship is due to mechanical forces associated with bottle-feeding or whether a reduction in, or lack of, breast milk intake or a lack of complimentary foods alongside breast milk results in nutritional differences that impact skull development [66]. As our study also highlighted an association between obesity and DP, research on other early-life or environmental exposures should be conducted to elucidate their effects on growth and development, particularly skull growth and head shape [67,68]. Finally, randomized controlled trials, although considered the gold standard study design for obtaining reliable evidence of an intervention’s effectiveness, should not be conducted for the interventions herein that relate to risk factors for DP development that have already been assessed through meta-analysis. However, randomized controlled trials could provide further evidence for an intervention—if ethically appropriate—where the evidence for a particular risk factor is relatively weak, such as when only a single cohort study has provided the evidence for a risk factor’s influence on the development of DP.

## Appendix

Multimedia Appendix 1 PRISMA 2020 checklist.

## Abbreviations

aOR: adjusted odds ratio
DDH: developmental dysplasia of the hip
DP: deformational plagiocephaly
GRADE: Grading of Recommendations Assessment, Development, and Evaluation
OR: odds ratio
PRISMA: Preferred Reporting Items for Systematic Reviews and Meta-Analyses
RR: risk ratio

## Data Availability

All data generated or analyzed during this study are included in this paper.
